# Late-Onset Masseteric Metastasis of Malignant Melanoma in a Patient With Neurofibromatosis and Lipomatosis: A Diagnostic Challenge and Case Report

**DOI:** 10.7759/cureus.50847

**Published:** 2023-12-20

**Authors:** Maria Inês Borges, João M Abreu, Fátima Ramalhosa, Simão Nogueira, Ana Corte Real

**Affiliations:** 1 Department of Stomatology, Clinical and Academic Centre of Coimbra, Coimbra, PRT; 2 Faculty of Medicine, Clinical and Academic Centre of Coimbra, Coimbra, PRT; 3 Department of Pathology, Clinical and Academic Centre of Coimbra, Coimbra, PRT

**Keywords:** palliative medicine, palliative care, lipomatosis, neurofibromatosis 1, metastasis, melanoma

## Abstract

Melanoma and neurofibromatosis (NF) are distinctly separate conditions, each characterized by unique pathophysiological processes. Nevertheless, their clinical presentations can share overlapping similarities. This report highlights a unique case involving a 68-year-old male with NF1 and lipomatosis, whose unwavering belief that a developing mass in the masseter region was benign and linked to the pre-existing diagnoses contributed to a significant delay in seeking healthcare. Consequently, this postponement resulted in the late diagnosis of disseminated malignant melanoma (stage IV, T4N0M1c). Given the patient's prognosis and poor general health, a palliative treatment plan was devised, entailing the complete excision of the masseteric mass and vertebral radiotherapy. Following a rapid and extensive progression of the cancerous lesions, the patient passed away in a palliative care infirmary four months after surgery. The significance of this case, justified not only by its uncommon presentation and atypical differential diagnosis, highlights the critical necessity of regular follow-up protocols for melanoma patients, particularly those prone to metastasis, while ensuring patient attendance. Furthermore, it underscores the necessity of patient education, particularly in recognizing early signs and symptoms, and timely intervention in cases with complex comorbidities.

## Introduction

Melanoma and neurofibromatosis (NF) are distinctly separate conditions, each characterized by unique pathophysiological processes. Nevertheless, on rare occasions, their clinical presentations can share overlapping similarities.

Responsible for 1.7% of global cancer diagnoses, melanoma originates from the malignant transformation of melanocytes [1,2]. With an extremely high propensity for metastasis, it positions itself as the most lethal skin cancer [1,2] and the second most lethal oncological disease [3].

Although melanoma can affect individuals of all ages, its highest incidence occurs around the age of 65, often attributed to cumulative exposure to UV radiation. Other risk factors include sunburns, indoor tanning, light hair and skin, multiple melanocytic naevi, and a family history of melanoma [1-4].

Characterized as a pigmented lesion, melanomas should be clinically evaluated according to the ABCDE algorithm. However, it is important to consider that even 5 mm or smaller lesions can potentially still be primary melanomas. Hence, while dermatoscopy is a fundamental diagnostic tool for pigmented lesions, the definitive diagnosis is confirmed via a biopsy [4]. Current guidelines also recommend BRAF testing in patients with stage III/IV melanoma, as well as stage IIC high-risk resected disease [4].

Clinical and histopathological aspects also play a pivotal role in classifying melanoma into its various subtypes, which include superficial spreading melanoma, nodular melanoma, lentigo maligna melanoma, acral lentiginous melanoma, ocular melanoma, mucosal melanoma, acral melanoma, spitzoid melanoma, and desmoplastic melanoma [2].

Treatment for early-stage melanoma often involves the complete surgical removal of the primary lesion with appropriate margins, yielding high survival rates [5]. For those contending with stage III or regional disease, studies highlight a substantial enhancement in patients' outlook through the integration of adjuvant therapies in conjunction with surgery [6]. Despite these advancements, the five-year survival rates for stage III melanoma patients remain notably diverse, ranging from 93% (IIIA) to 32% (IIID) [7]. Nonetheless, even at their lowest, these rates signify a substantially better prognosis compared to cases of disseminated disease, where survival rates plummet to 23% or lower [3].

NF1 and 2 and schwannomatosis represent a group of hereditary syndromes characterized by dominant autosomal transmission, leading to a predisposition for both benign and malignant tumors [8].

Standing out as the most prevalent form, NF1 encompasses 90% of cases and takes place due to a gene mutation at 17q11.2 [8]. Clinical manifestations include distinctive features such as "Café-au-lait" spots, axillary and inguinal freckles (Crowe sign), cutaneous and/or plexiform neurofibromas, and Lisch nodules. The development of malignant tumors impacting both the peripheral and central nervous systems, such as gliomas and malignant peripheral nerve sheath tumors, can also occur. Additional signs may include individual cognitive and/or behavioral changes, skeletal anomalies, and increased susceptibility to various cancers, in particular gastrointestinal tumors, breast cancer, leukemia, and neuroendocrine tumors [9]. Oral manifestations are also common in NF1, with nodular neurofibromas frequently impacting the tongue, buccal mucosa, and/or lips. Enlarged fungiform papillae, sometimes conducting to macroglossia, are also a common finding [10].

NF1 diagnosis is based on clinical criteria established by international consensus and requires the identification of at least two pre-established characteristics. However, if a direct relative has a confirmed diagnosis of NF1, only one characteristic is necessary for diagnosis [11].

Treatment in patients with NF1 typically follows a symptom-oriented approach, thereby limiting therapeutic intervention to a small fraction of cases. These include larger cutaneous neurofibromas, whether for aesthetic or medical reasons like pain and inflammation, as well as other benign or malignant tumors such as subcutaneous and plexiform neurofibromas or malignant peripheral nerve sheath tumors [12]. More recently, targeted therapies have emerged as a promising alternative for inoperable, highly symptomatic, and/or oncological NF1 patients [8].

Despite these advancements, individuals with NF1 continue to experience substantial morbidity and a 10- to 15-year decreased life expectancy compared with the general population [13].

This study details the unique case of a 68-year-old male presenting a late recurrence of melanoma, initially characterized by a masseteric metastasis, whose healthcare seeking and referral were delayed due to the previous diagnoses of benign conditions such as NF1 and lipomatosis. A summarized version of this case was previously presented as a meeting abstract and poster at the “XXXVI Annual Congress of the Portuguese Society of Stomatology and Dental Medicine” on June 8, 2016.

## Case presentation

A 68-year-old male farmer diagnosed with NF1 and lipomatosis sought an appointment at a public health institution's outpatient department for evaluation of a tumoral mass in the left masseteric region. During consultation, the patient described the mass as painless and progressively increasing in size over the preceding six months. The patient also clarified that healthcare wasn’t sought earlier due to his strong conviction that the mass was benign in nature and associated with NF1 or lipomatosis. Additionally, asthenia, unquantified weight loss requiring a two-belt-hole reduction, and moderate back pain conditioning a limitation of spinal movements were also reported. No additional signs or symptoms were described.

The patient's medical history included substantial sun exposure and a prior diagnosis of hypertension. Furthermore, the patient's surgical history encompassed an oropharyngeal plexiform neurofibroma excision one year earlier. Additionally, five years prior, the patient underwent surgery at a different healthcare facility for a stage IIB (T4aN0M0) malignant melanoma on the nasal bridge. However, follow-up was discontinued due to the patient relocating to a different city and facing significant challenges in attending consultations.

**Figure 1:**
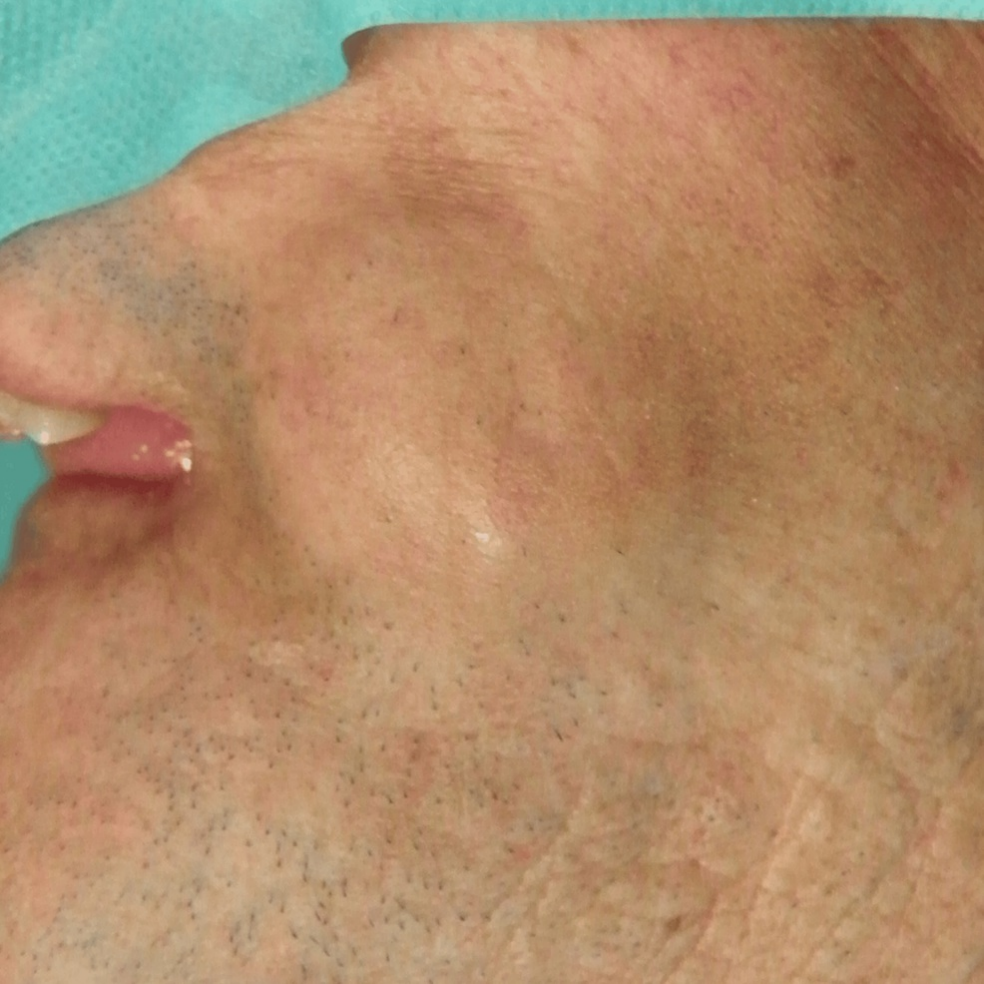
Subcutaneous masseteric mass Visible in the facial left lateral view is a subcutaneous mass, situated in the vicinity of the left masseter muscle.

**Figure 2:**
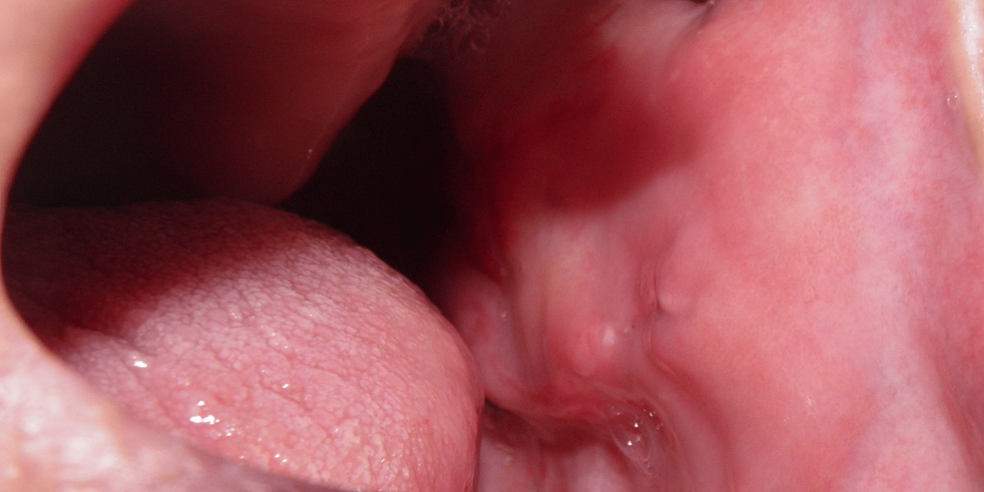
Intraoral projection of the masseteric mass Within the intraoral left view, a submucosal mass is identified, occupying a central position in the buccal mucosa.

**Figure 3:**
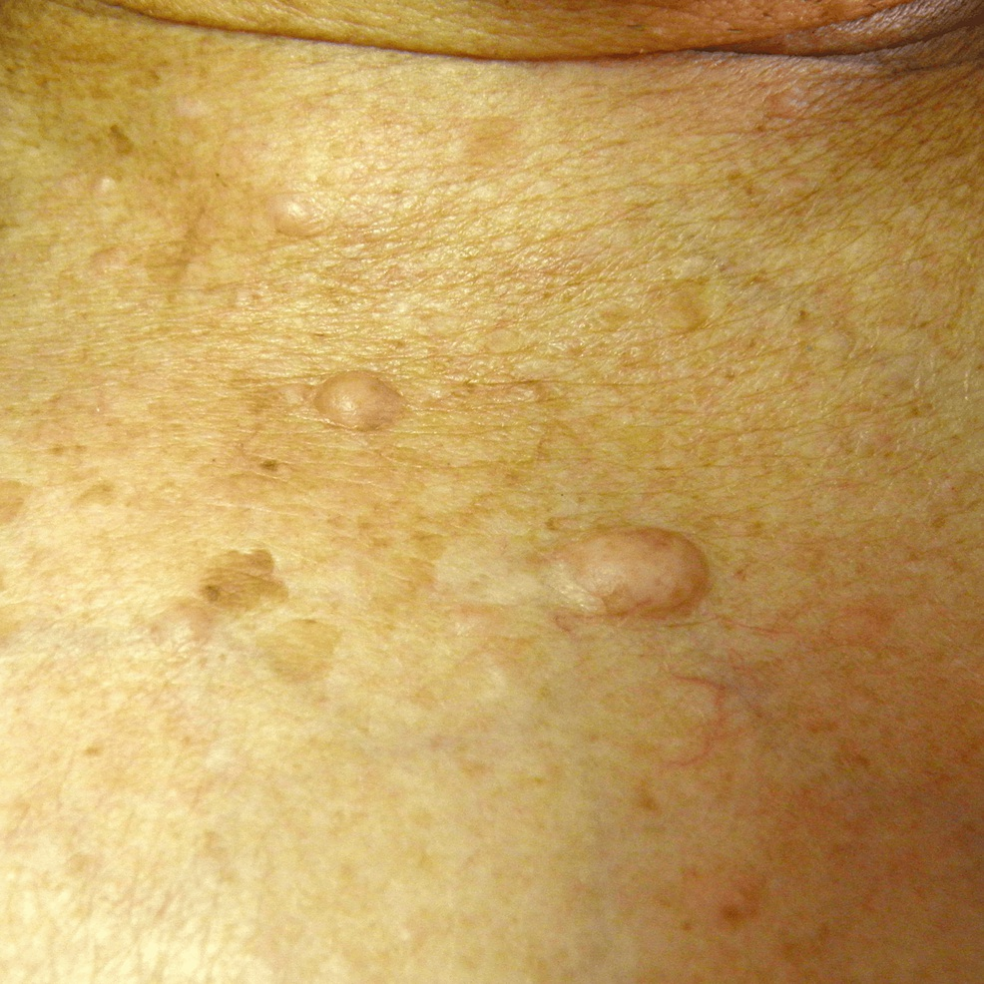
Cutaneous neurofibromas Displayed in a semi-frontal view are two cutaneous neurofibromas situated on the left upper chest area, measuring approximately 0.5 cm and 1 cm in width, respectively.

**Figure 4:**
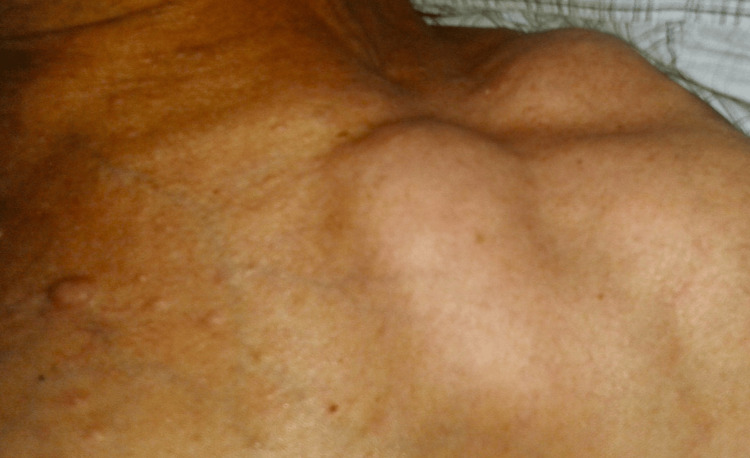
Subcutaneous lipomas Visible in a 45-degree angled view are two subcutaneous tumoral projections situated within the left supraclavicular area, measuring approximately 3 and 4 cm in width, respectively.

Clinical examination confirmed the existence of a nodular mass in the left masseteric region (Figure 1), projecting into the buccal mucosa (Figure 2), approximately 40 mm in width. Physical inspection also revealed the presence of multiple cutaneous neurofibromas (Figure 3) and two established left supraclavicular masses previously diagnosed as lipomas (Figure 4). The palpation of the masseteric lesion revealed a firm texture, irregular borders, and attachment to deeper tissues. There were no detectable cervical or axillary lymph node enlargements upon the exploration of said areas.

**Figure 5:**
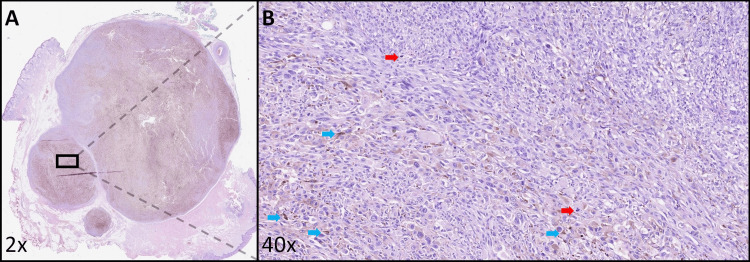
Metastatic malignant melanoma (histological analysis with hematoxylin and eosin staining) A: At a 2x optical magnification, tumoral nodules characterized by confluent growth and well-defined boundaries are evident. These nodules are demarcated on one side by oral mucosa and on the contralateral side by cutaneous epithelium. Notably, the nodules exhibit abundant melanocytic pigmentation. B: Observed at a 40x optical magnification, the metastatic melanoma deposit manifests as a collection of large atypical epithelioid cells exhibiting robust growth in solid sheets. These cells demonstrate distinctive cytological atypia, marked pleomorphism, heightened proliferative activity (red arrows), and conspicuous intracytoplasmic pigmentation (blue arrows). Colored arrows have been placed for illustrative purposes, highlighting representative elements within the image.

**Figure 6:**
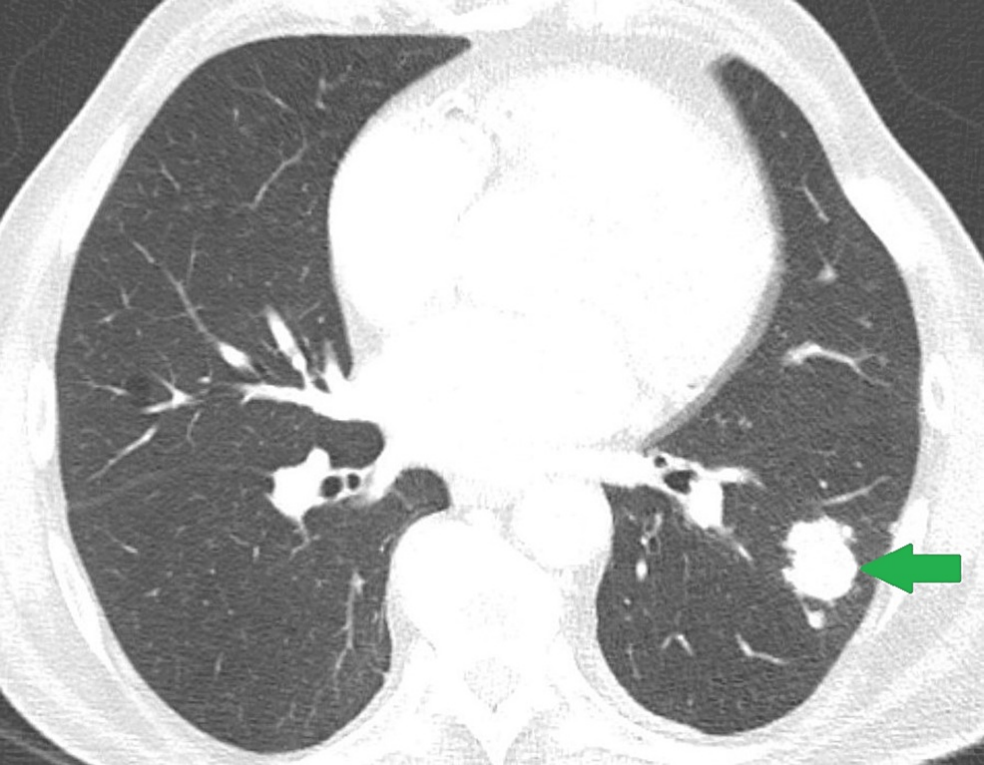
Pulmonary metastasis Within the axial view of the thoracic CT scan, a distinct non-calcified nodular lesion (green arrow) measuring 3 cm in width is visible in the lower left pulmonary lobe. Its morphological characteristics and location suggest the possibility of a metastatic lesion.

**Figure 7:**
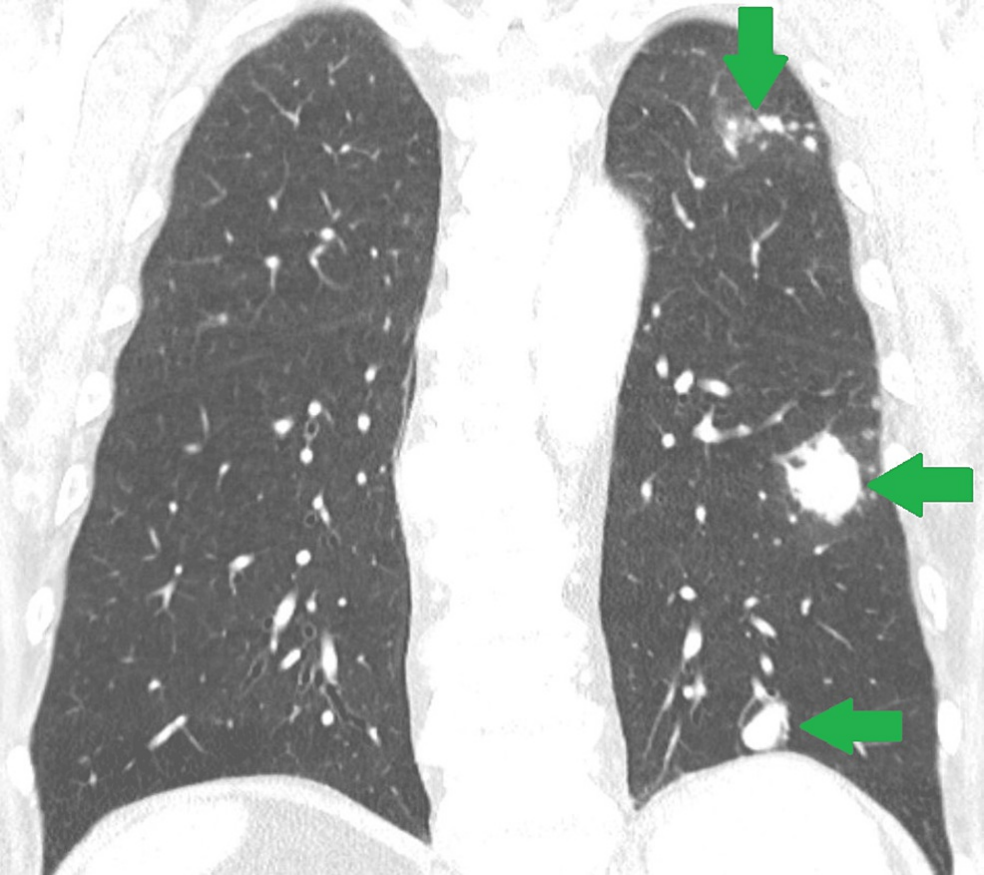
Pulmonary metastasis Within the coronal view of the thoracic CT scan, two distinct nodular lesions (horizontal green arrows), measuring 3 cm and 2 cm in width, respectively, are visible in the lower left pulmonary lobe. Moreover, smaller infracentimetric nodules are also present in the upper left pulmonary lobe (vertical green arrow). The imaging characteristics of these masses are indicative of metastatic lesions.

**Figure 8:**
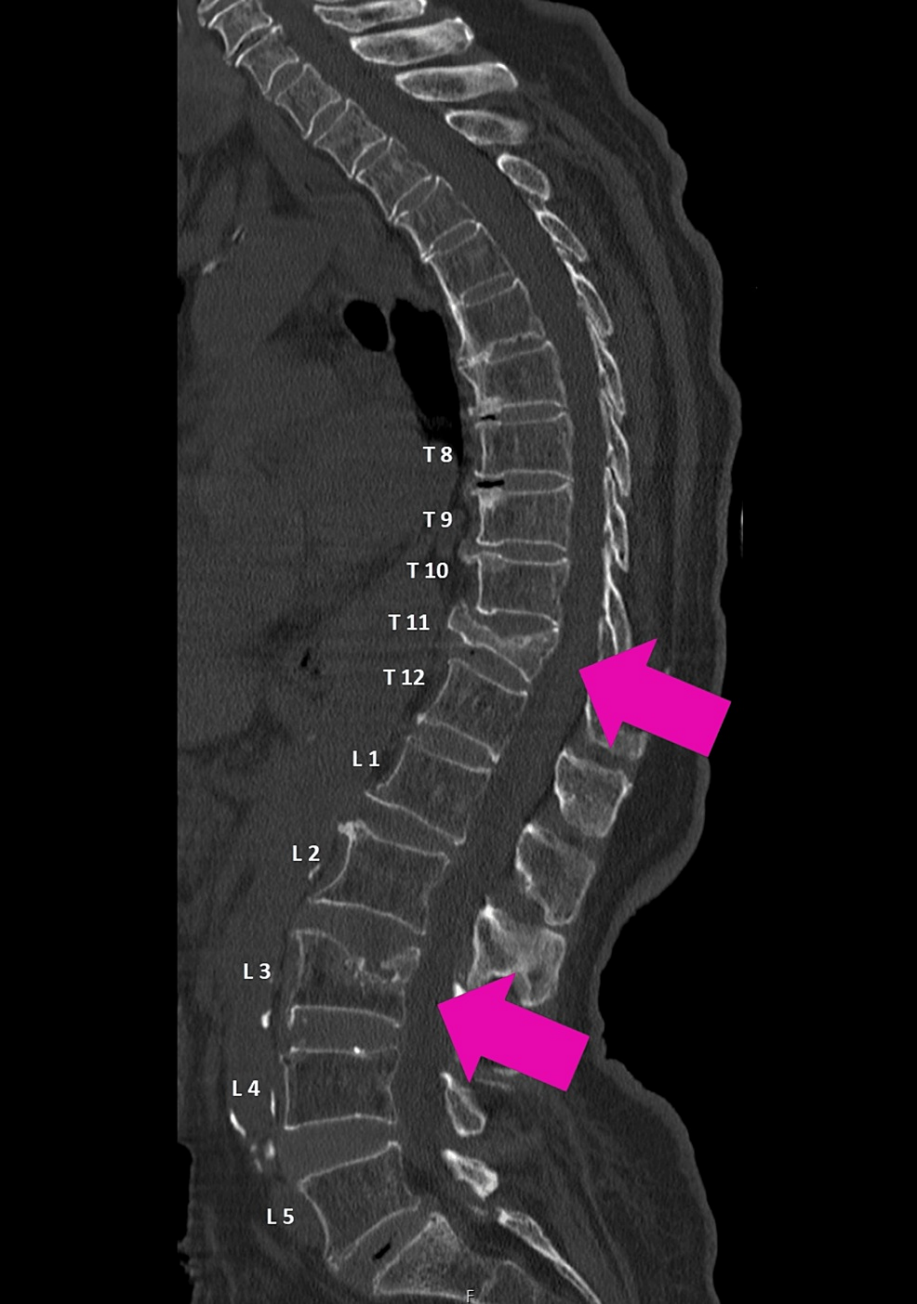
Vertebral metastasis Visible in the spine CT scan sagittal view, evidence of vertebral body collapse at T11 (upper pink arrow) and superior vertebral body irregularity at L3 (lower pink arrow) has been noted, consistent with pathological fractures attributable to metastatic lesions.

For diagnostic purposes, the patient was informed about and consented to undergo an incisional biopsy of the mass. During the procedure, macroscopically, an intense black pigmentation was visible, supporting the diagnostic hypothesis of malignant melanoma, a finding later corroborated through histological analysis (Figure 5). Following confirmation, a BRAF mutation test was conducted, yielding a negative result. A complementary study with a maxillofacial and cervical-thoracic-abdominal and spinal CT scan was also performed, identifying multiple nodular masses in the lungs (Figures 6-7) and pathological vertebral fractures (Figure 8), compatible with metastatic lesions.

**Figure 9:**
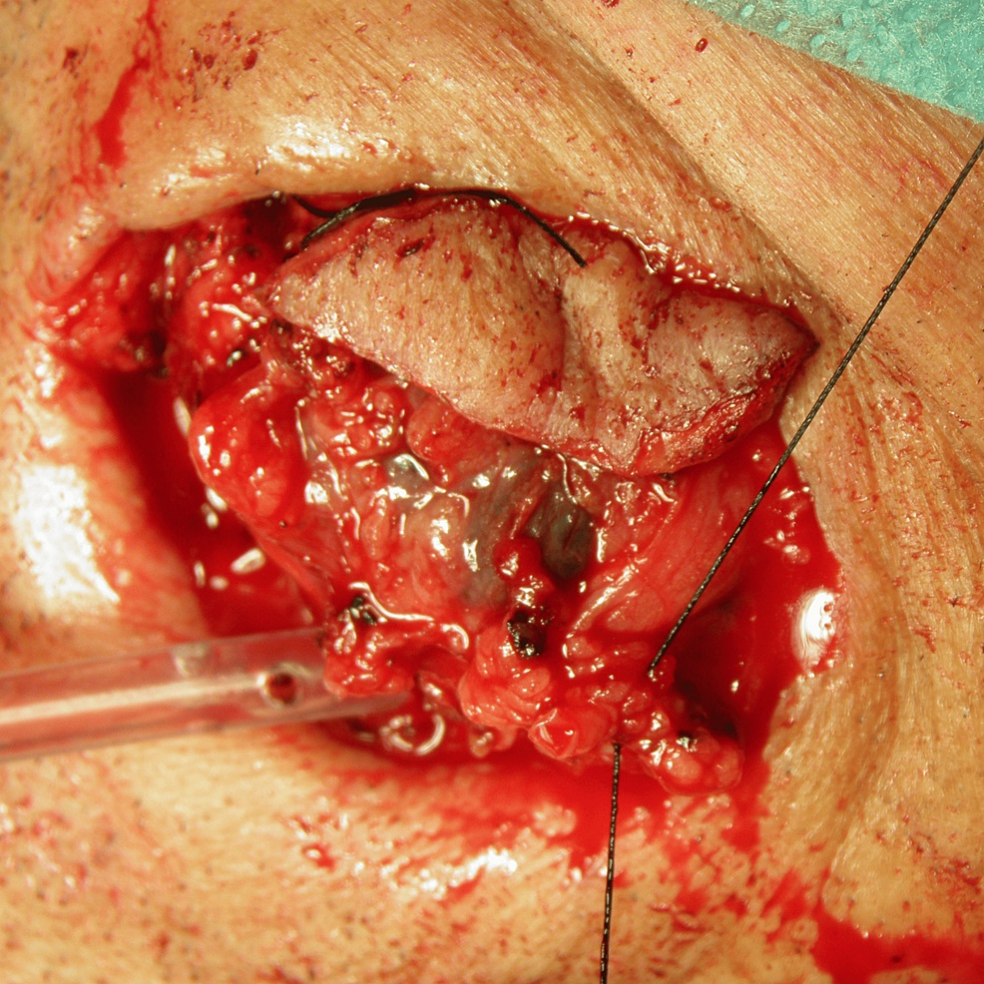
Masseteric mass excision Surgical excision of the melanoma metastasis.

**Figure 10:**
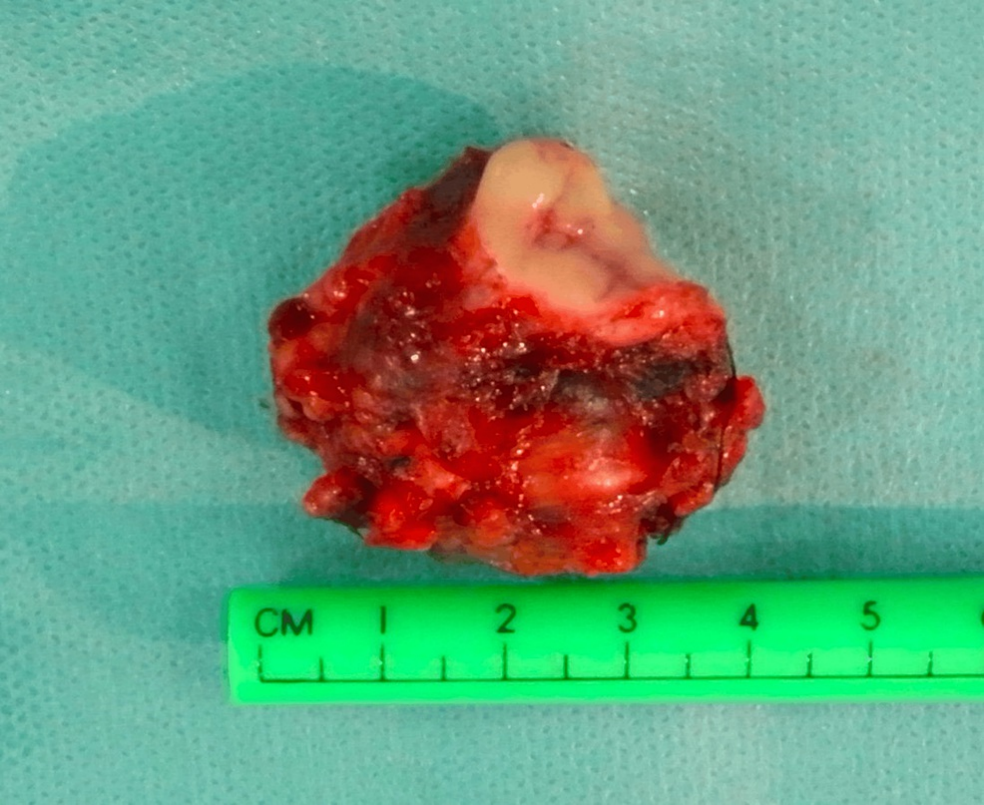
Masseteric mass excision Post-excision view of the melanoma metastasis.

Classified as stage IV malignant melanoma (T4N0M1c), the patient's case was presented in a multidisciplinary team meeting, and a treatment plan involving the complete excision of the masseteric mass (Figures 9-10) and palliative radiotherapy for the vertebral metastases was executed.

Following the rapid and extensive progression of the cancerous lesions, the patient was transferred to a palliative care infirmary one-month post-surgery, where he passed away within three months.

## Discussion

Manifesting as a notably aggressive cancer, up to 85% of malignant melanoma metastasis occurs within the initial three years following diagnosis [14], typically spreading to various skin areas, subcutaneous tissue, and organs such as the lungs, liver, bones, and brain [15]. Late masseteric metastasis as the primary indication of metastatic disease is exceedingly rare [16].

Moreover, various systemic diseases can also exhibit oral and maxillofacial symptoms, such as facial nodular masses [10,17], thereby creating a diagnostic challenge and eventually delaying a definitive diagnosis due to the confounding similarity and overlap of presentations.

The aforementioned scenario unfolded in the present case, where the patient's documented history of melanoma was overlooked in favor of benign entities such as NF1 and lipomatosis. Nevertheless, upon a comprehensive comparison of the clinical features of the lesion with the characteristics of the diagnostic hypotheses, the inclination tended toward a malignant or an aggressive benign entity.

This hypothesis is attributed to the hard consistency, irregular borders, adherence to the deep planes, and rapid and considerable growth of the presented masseteric mass, antagonistic features to the soft, well-defined, mobile, slow-growing, and painless lesions that depict the cutaneous lipomas present in a disease such as lipomatosis [17]. Neurofibromas, on the other hand, are characterized by their clinical heterogeneity, making them capable of presenting virtually identically to the said entity and therefore being confounded with a cancerous lesion [18].

After confirming the diagnosis of melanoma recurrence through histopathological analysis, further diagnostic evaluations were conducted, uncovering lung and vertebral metastasis, both of which are well-documented in the literature [15]. With the disease classified as stage IV (T4N0M1c), the patient was assessed in a multidisciplinary team consultation, enabling the decision to initiate palliative treatment, considering the patient’s general health, disease progression, and prognosis [4]. The plan included local control through the excision of the masseteric mass and vertebral radiotherapy. No therapeutic approach was conceived for the lung metastasis due to their extent and locations.

The excision of a melanoma lesion was initially conducted in 1787 by Dr. John Hunter, and, since then, it has become the primary treatment for localized primary melanoma. A sentinel lymph node biopsy is advised for tumors thicker than 0.8 mm or those that are ulcerated (stage pT1b or higher), with subsequent lymph node dissection typically recommended upon detection of melanoma cells in the sentinel node or the presence of clinical or imaging evidence of adenopathy [5]. However, there is a shifting landscape in surgical practice for patients with regional melanoma, with recent evidence suggesting that conducting radical lymph node dissection after a positive sentinel lymph node excision may not provide a survival advantage [6].

A conservative surgical approach, particularly metastasectomy surgery, may also be considered as a therapeutic option to minimize widespread disease impact [19,20], along with locoregional radiotherapy, particularly when the patient's general status contraindicates a more aggressive intervention, as in the presented case [4]. This methodology allows the relief and/or prevention of specific symptoms or complications, helping to control the disease's effect in a predetermined area and slowing down the progression, potentially offering a survival advantage [19,20].

For unresectable stage III and IV lesions, therapeutic options, including immunotherapy and targeted therapies, are currently available. Despite these advancements, the prognosis for these patients remains grim, with only one in five individuals with disseminated disease surviving beyond five years [3]. Nevertheless, one should consider them as valid alternatives, particularly when combined with surgery, chemotherapy, and/or radiotherapy, since studies have shown increased survival [3,15]. In this case, none of these options were implemented due to the patient’s rapid deterioration of his general condition and BRAF negativity.

Following a rapid and extensive progression of the cancerous lesions, the patient passed away in a palliative care infirmary four months after surgery, an expected outcome and in agreement with existing literature [4,5].

## Conclusions

The significance of this case, justified by its uncommon presentation and atypical differential diagnosis, highlights the critical necessity of regular follow-up protocols for melanoma patients, particularly those prone to metastasis, while overcoming various difficulties and ensuring patient attendance. Furthermore, it underscores the necessity of patient education, particularly in recognizing early signs and symptoms, and timely intervention in cases with complex comorbidities, improving survival rates in individuals with melanoma.
